# Utility of Glycosylated TIMP3 molecules: Inhibition of MMPs and TACE to improve cardiac function in rat myocardial infarct model

**DOI:** 10.1002/prp2.442

**Published:** 2018-11-14

**Authors:** Vishnu Chintalgattu, Joanne Greenberg, Shivani Singh, Venice Chiueh, Amy Gilbert, Jason W. O'Neill, Stephen Smith, Simon Jackson, Aarif Y. Khakoo, TaeWeon Lee

**Affiliations:** ^1^ Cardiometabolic Disorders & Therapeutic Discovery Amgen Discovery Research South San Francisco California

**Keywords:** ECM degradation, glycosylation, myocardial infarction, TIMP3

## Abstract

Tissue Inhibitor of Metalloproteinase 3 (TIMP3) is a secreted protein that has a great utility to inhibit elevated metalloproteinase (MMP) activity in injured tissues including infarcted cardiac tissue, inflamed vessels, and joint cartilages. An imbalance between TIMP3 and active MMP levels in the local tissue area may cause worsening of disease progression. To counter balance elevated MMP levels, exogenous administration of TIMP3 appeared to be beneficial in preclinical studies. However, the current form of WT‐TIMP3 molecule has a limitation to be a therapeutic candidate due to low production yield, short plasma half‐life, injection site retention, and difficulty in delivery, etc. We have engineered TIMP3 molecules by adding extra glycosylation sites or fusing with albumin, Fc, and antibody to improve pharmacokinetic properties. In general, the C‐terminal fusion of TIMP3 improved expression and production in mammalian cells and extended half‐lives dramatically 5‐20 folds. Of note, a site‐specific glycosylation at K22S/F34N resulted in a higher level of expression and better cardiac function compared to other fusion proteins in the context of left ventricle ejection fraction (LVEF) changes in a rat myocardial infarction model. It appeared that cardiac efficacy depends on a high ECM binding affinity, in which K22S/F34N and N‐TIMP3 showed a higher binding to the ECM compared to other engineered molecules. In conclusion, we found that the ECM binding and sustained residence of injected TIMP3 molecules are important for cardiac tissue localization and inhibition of adverse remodeling activity.

**Abbreviations**
ECM: extracellular matrix
HCF: human cardiac fibroblast
LAD ligation: left anterior descending artery ligation
LRP‐1: low density lipoprotein receptor‐related protein‐1
LVEF: left ventricle ejection fraction
MI: myocardial infarction
MMP: metal metalloproteinase
TACE: TNFα converting enzyme
TIMP3: Tissue inhibitor of metalloproteinases subtype 3

## 1 INTRODUCTION

The extracellular matrix (ECM) is an important biological component for structural and biochemical reactions to the surrounding cells. The ECM turnover is a tightly regulated process that maintains a balance between matrix metalloproteinases (MMPs) and their endogenous inhibitor Tissue Inhibitor of Metalloproteinases (TIMPs) levels. It has been suggested that blocking MMP activity either by small molecule inhibitor or TIMP molecules immediately after disease‐causing insults would be beneficial for various disease modifying conditions including acute myocardial infarction, acute lung injury, osteoarthritis, and inflammation.^1^, ^2^, ^3^, ^4^ However, small molecule MMP inhibitors have failed in a clinical heart failure trial due to intolerable side effects, particularly musculoskeletal pain syndrome, which could be due to metabolites or off‐target effects.^5^, ^6^, ^7^ There have been many trials of more specific inhibitors to avoid this side effect without resolution.^8^ Cardiac tissues in response to ischemia induce the breakdown of ECM by elevating MMP levels, which induces ventricular wall thinning in the infarcted area eventually resulting in heart failure.^9^, ^10^ Postischemic loss of cardiomyocytes incites inflammatory cells, mostly neutrophils, to spike MMP levels disproportionally at the infarct site. Inadequate levels of TIMP3 to counteract protease activity at infarct site results in extensive ECM remodeling, ventricular dilation, and decline in cardiac function.^11^, ^12^ Inhibition of MMPs, specifically MMP2 and MMP9, has been shown to prevent ECM proteolysis and reduce the infarct size following myocardial injury.^13^, ^14^

All mammalian TIMPs (TIMP1‐4) consist of two domains: N‐terminal and C‐terminal, with the N‐terminal domain, designated N‐TIMP exhibiting function. Each TIMP subtype shows inhibition of a distinct subset of MMPs, with TIMP3 being the broadest MMP, ADAM, and ADAM‐T inhibitor.^15^ TIMP3 is the most abundant subtype in the heart, and inhibits post‐MI remodeling by reducing TNFα production via TACE (TNF alpha converting enzyme) inhibition as well as by decreasing endogenous MMP activity in the infarcted area.^7^, ^16^ These dynamic changes of upregulated MMPs and decreased TIMP3 levels in myocardial matrix following MI lead to myocardial ECM degradation contribute to cardiac dysfunction and adverse remodeling in the failing heart.^17^, ^18^ The level of TIMP3 was significantly reduced in post‐MI animals and patients with dilated cardiomyopathy.^19^, ^20^ Thus, it is hypothesized that the exogenous administration may compensate for the loss of cardiac tissue TIMP3 after myocardial injuries.

Modification by engineering of large protein molecules is a common method to extend serum half‐life. The most common modifications are fusion with albumin or Fc, which significantly extended the half‐life of protein ligands, such as interferon gamma (γINF)‐fused with human serum albumin.^21^ PEGylation (polyethylene glycol conjugation) has a significant pharmacokinetic effect by slowing down clearance, however there seems to be some safety concerns regarding vacuolization in renal cells.^22^, ^23^ An additional modification approach is a site‐specific glycosylation of protein molecules that improves pharmacokinetic (PK) property by increasing stability due to reduced protease sensitivity with bulky glycol moiety, which may reduce potential immunogenicity as well.^24^, ^25^ The serum half‐life and in vivo bioactivity of glycosylated recombinant human erythropoietin (rHuEPO or darbepoetin alfa), which is used to treat anemia, was improved 3‐fold upon glycosylation of two additional sites (5‐N‐Glyco and 1‐O‐Glyco) of recombinant EPO protein.^26^

TIMP3 is a high affinity ECM binding protein. The ECM binding motif of TIMP3 has been characterized previously and shows that both N‐ and C‐terminal domains interact with the ECM via positively charged Lys and Arg residues.^27^ The N‐terminal domain amino acid residues 1‐120 is biologically active and can inhibit both MMP and TACE activity, although the inhibitory mechanisms may be distinct as revealed by mutations and crystal structures.^28^, ^29^ TIMP3 is a known substrate for the endocytic receptor called the low density lipoprotein receptor‐related protein‐1 (LRP‐1). Blocking LRP‐1 interaction may reduce clearance and extend systemic half‐life.^30^

In this study, we have engineered TIMP3 molecules to improve protein expression, serum half‐life, and its functional consequences. We have explored glycosylation, PEGylation, and fusion (Fc, HSA, and Ab) modifications and characterized biological properties including activity as well as ECM binding capability. Modified TIMP3 proteins maintained in vitro MMP activity with somewhat lower activity with the bulkiness of fusion partners, especially with 150 kDa antibody fusion. Of interest, site‐specific glycosylation by K22S/F34N TIMP3 (v2) significantly increased expression compared to the WT‐TIMP3 with similar short serum half‐life (<1 hour). A mutant with multiple glycosylation sites (H55N/Q57T/K71N/E73T/D87N/K89T/R115T, v82) retained MMP2/MMP9 and TACE inhibitory activity, but extended serum half‐life approximately 5‐fold (~4 hours) compared to the WT‐TIMP3 or the glycovariant TIMP3v2 molecule in rat studies. Further C‐terminal fusion of TIMP3v2 with human serum albumin, Fc, or heavy chain or light chain antibody extended serum half‐life (2.7 hours, 23.1 hours, 2.5 hours, 7.5 hours, respectively) in rats. Additionally, the PEGylation (20 kDa) of TIMP3v2 significantly extended the serum half‐life (28.6 hours). In vivo cardiac function (%EF) was improved with the TIMP3 treated compared to the untreated at Day 7 after administration in the rat MI model when it was delivered directly into myocardium after myocardial infarct induction. However, cardiac functional improvement was less significant when TIMP3 was delivered via intravenous injection compared to the direct myocardial injection scheme. It was assumed that the ECM binding activity might play a critical role in a sustained MMP inhibitory activity. Interestingly, TIMP3v2 and N‐TIMP3 exhibited the highest ECM binding affinity from the washout experiment. On the contrary, highly glycosylated and C‐terminal fusion molecules did not bind to the ECM with high affinity.

## 2 MATERIALS AND METHODS

### 2.1 Production of TIMP3 variants

Human TIMP3, modified TIMP3 with glycosylation mutations (v2, v82), and fusion constructs (HSA, Fc, or Ab) were expressed in a Chinese hamster ovary cell line, whereby conditioned media were concentrated by tangential flow filtration (TFF; Millipore,10 kDa MWCO) and filtered. The TIMP3 and fusion constructs were next purified through a three‐column chromatography procedure, each utilizing a specific capture column; TIMP3 and glycosylation mutants with Capto MMC (GE Healthcare, Freiburg, Germany): HSA fusions with Cibacron Blue (Merck KGaA, Darmstadt, Germany): and Fc or Ab fusions with MabSelect Sure (GE Healthcare, Pittsburg, PA). Each of these was then followed by Capto Adhere and SP HP (GE Healthcare, Freiburg, Germany) chromatography steps. The TIMP3 containing SP HP fractions were finally concentrated and buffer exchanged (10 mmol/L sodium acetate pH 5.2, 9% sucrose) by TFF. N‐TIMP3 (13.9 kDa N‐domain; aa 1‐120) was expressed as inclusion bodies in an *E. coli* system with a subtilisin prodomain fusion. After refolding and prodomain cleavage, the N‐TIMP3 was purified by a three‐column procedure using SP HP, CHT, and Butyl HP chromatography. The N‐TIMP3 containing Butyl HP fractions were also concentrated and buffer exchanged (10 mmol/L sodium acetate pH 5.2, 9% sucrose) by TFF (Pall, 5 kDa MWCO).

### 2.2 Cell Culture of human cardiac fibroblasts, preparation of cell free matrix, and ECM retention assay

Human cardiac fibroblasts (HCFs, PromoCell) were cultured in fibroblast growth media (FGM‐3, Lonza) supplemented with βFGF, insulin, GA‐1000, and 10% fetal bovine serum at 37°C containing 5% CO_2_ and trypsinized every 5 days. For decellularization, HCFs were grown on collagen I coated 96‐well plate (Biocoat, Corning) for 7‐10 days before decellularizing in 200 μL lysis buffer (8 mmol/L Na_2_HPO_4_, 1% NP‐40, pH 9.6) at 37°C for 15 minutes after washing with wash buffer I (100 mmol/L Na_2_HPO_4_, 2 mmol/L MgCl_2_, 2 mmol/L EGTA, pH 9.6). The wells were further incubated with lysis buffer for 75 minutes at 37°C and washed twice with 200 μL wash buffer II (300 mmol/L KCl, 10 mmol/L Na_2_HPO_4_, pH 7.5) and dH_2_O. Decellularized wells were stored in PBS at 4°C until use.

To visualize decellularized wells by immunostaining, the wells were fixed with 4% paraformaldehyde in PBS for 15 minutes and stained using rabbit anti‐fibronectin (Abcam, 1:200), rabbit anti‐Collagen type I (Abcam, 1:50), and rabbit anti‐Collagen type III (Abcam, 1:200) primary antibodies in 2% BSA. The wells were stained with Alexa Fluor 488 conjugated goat anti‐rabbit and goat anti‐mouse secondary antibodies in 2% BSA‐PBS (Invitrogen, 1:200). The immunostained wells were visualized using EVOS fluorescent microscope (Life technologies). Deposition of ECM fibrils in decellularized wells was quantified from the collected images as percentage of area positively stained with anti‐fibronectin using ImageJ software.

For ECM binding of engineered TIMP3 variants, decellularized ECM in 96‐well plates (16 000 cells/well) was incubated with TIMP3 in 100 μL PBS and 0.1% BSA at RT. IR800‐labeled TIMP3 or anti‐TIMP3 antibodies were employed to detect ECM bound TIMP3. The bound IR (infrared) signal was measured using an Odyssey IR imager (LI‐COR Biotechnology). Unlabeled TIMP3 was incubated with ECM and fixed in 5% paraformaldehyde before and after washes with 100 μL PBS. The fixed wells were incubated with anti‐TIMP3 C‐terminus (Millipore, 1:500) or, anti‐TIMP3 N‐terminus (Abcam, 1:200) antibodies, and IR800‐labeled goat anti‐rabbit (LI‐COR, 1:200) secondary antibodies in 2% BSA‐PBS, followed by measurement on Odyssey imager.

### 2.3 MMP and TACE activity assay

MMP activity was measured by using fluorimetric methods. Fluorescence signal is increased upon cleaving a quenched MMP subtype specific 5‐FAM/QXL 520 fluorescence resonance energy transfer (FRET) peptide substrate (Anaspec, Fremont, CA) by an activated MMP subtype or subtype specific catalytic domain. For MMP2 activity assay, recombinant human pro‐MMP2 (Anaspec, Fremont, CA) and rat pro‐MMP2 (R&D systems) are activated with 1 mmol/L 4‐aminophenylmercuric acetate (APMA, Anaspec, Fremont, CA) for 1 hour at 37°C before incubating with MMP2 sensitive 5‐FAM/QXL 520 FRET peptide in assay buffer provided by the vendor against various concentrations of TIMP3 in a black 384‐well Optiplate (PerkinElmer, Waltham, MA) at 37°C. After 90 minutes incubation, fluorescence signal from the reaction plate was measured at excitation (490 nm) and emission (520 nm) on EnVision multilabel microplate reader (PerkinElmer, Waltham, MA). For MMP9 activity measurement, a catalytic domain of human MMP9 (Anaspec, Fremont, CA) or rat pro‐MMP9 (R&D systems) was incubated with MMP9 sensitive 5‐FAM/QXL 520 FRET peptide and various concentrations of TIMP3 in a black 384‐well Optiplate (PerkinElmer, Waltham, MA) at 37°C. Data in relative fluorescence unit (RFU) are plotted against tested TIMP3 concentrations in GraphPad Prism 5.0 (GraphPad, San Diego, CA) to estimate half maximal inhibition constant (IC_50_). TACE assay was performed by a fluorometric assay kit (SensoLyte 520 TACE α‐secretase, Anaspec, Fremont, CA) similar to the above MMP assay according to the manufacturer's instruction. Bioactivity of ECM‐bound TIMP3 was determined by the above MMP2 and MMP9 assays. The decellularized ECM was gently washed thrice with 100 μL of PBS and incubated in 100 μL of assay buffer containing 25 ng of purified human MMP2 or MMP9 and quenched fluorescent peptide substrate diluted 1:400 for 90 minutes at 37°C. The signal at excitation/emission wavelengths 485 nm/520 nm was measured by fluorescence microplate reader EnVision (Perkin Elmer).

### 2.4 Gelatin zymography

Zymography is a conventional method for detecting MMP activity. To confirm MMP inhibition in gelatin zymography we used purified human MMP2 or MMP9 enzyme. After running the zymogram with human MMP2, the gel lanes were cut and incubated with engineered TIMP3 variants during development in a separate container overnight at 37°C before staining. For tissues, cardiac LV tissues were homogenized in RIPA Buffer Solution (Teknova, Hollister, CA) supplemented with PMSF. Tissue lysate (50 μg) or recombinant human MMP2, or MMP9 (10 ng, R&D Systems, Minneapolis, MN) was loaded per lane onto Novex 10% Zymogram Plus (Gelatin) Protein Gel (Thermo Fisher Scientific, Waltham, MA). After electrophoresis, gels were incubated with Novex Renaturing buffer (Thermo Fisher Scientific). Gels were then incubated overnight in Developing buffer (Thermo Fisher Scientific) with or without TIMP3 molecules. The following day, gels were stained with SimplyBlue Safestain (Thermo Fisher Scientific). MMP activity was quantified using ImageJ software.

### 2.5 Pharmacokinetic analysis in rats

Pharmacokinetics of TIMP3 (3 mg/kg, intravenous injection) was measured in normal male Sprague Dawley rats according to the IACUC approved protocol. TIMP3 concentrations were quantified from plasma using mass spectrometry analysis for nonfused molecules (LOQ: 10 ng/mL) and immunoassay (Gyrolab fluorescent assay, 10A7 mAb for capture and anti‐HSA or Fc for detection; LOQ 100‐250 ng/mL) for fused molecules. Plasma concentration data following IV administration were analyzed using either standard noncompartmental analysis or a constant IV infusion model in Phoenix version 6.4 (Pharsight, Mountain View, CA).

### 2.6 Echocardiography in MI rats

Myocardial infarction model was created by permanently ligating the left anterior descending artery of Sprague Dawley rats (Charles River Labs). After 3 hours of ligation, TIMP3 molecules (N‐TIMP3, v2 and v82) were injected directly into the infarcted myocardium at three separate injection sites with 31G needle under anesthesia. For infusion, TIMP3v82‐Fc (5 mg/kg, single IV bolus injection) was infused via tail vein injection post‐LAD ligation (3 hours) in SD rats before echo scanning at Day 7. Echocardiographic studies were performed under light anesthesia in 2% isoflurane with continuous monitoring of body temperature, blood pressure, and heart rate. LV end diastolic volume (LVEDV) and systolic volume (LVESV) were measured by two‐dimensional guided B‐mode echocardiography from long axis view using VisualSonics Imaging System (Vevo 2100) by applying sonography gel on the thorax. Ejection fraction (EF) was calculated with Vevo software and plotted. All in vivo experiments were conducted by the Institutional IACUC approved protocol in an AAALAC accredited facility.

### 2.7 Inhibition of extracellular matrix degradation

Degradation of ECM by collagenase type I (C2674 Sigma) was examined in presence of MMP inhibitors. ECM formed by 16000 HCF cultured for 7 days was used for degradation study. 100 μg of collagenase was incubated with TIMP3 or marimastat (Sigma‐Aldrich) in decellularized ECM in 96‐wells for 1 hour at 37°C with gentle shake. The wells were washed once with 100 μL of PBS, fixed with 4% paraformaldehyde for 15 minutes and immunostained for collagen type III.

### 2.8 Fluorescent labeled TIMP3 retention in MI rat hearts

To estimate ex vivo signals of cardiac residence half‐life, AF680‐labeled N‐TIMP3 (70 μg) or AF680‐labeled F‐TIMP3 (270 μg) was directly injected into the infarct area of MI rats at 1 hour post‐LAD ligation before closing the chest. Hearts were harvested at desired time points within 14‐day postinjection, and sliced 1 mm section on slicing block before putting into microplate, which was read in Safire multimode plate reader (Tecan) at 680 nm excitation and 702 nm emission wavelength.

### 2.9 Statistical analyses

For in vitro statistical analyses, data are reported as mean ± SD or SEM. Comparisons were made by paired one‐tailed Student *t* test between control and TIMP3 treated groups for at least three experimental data sets. For in vivo statistical analyses, comparisons were performed by two‐tailed Student *t* test between vehicle and TIMP3 treated groups. *P *<* *0.05 was considered statistically significant.

## 3 RESULTS

### 3.1 In vitro potency of MMP2/9 and TACE inhibition

**Figure 1:**
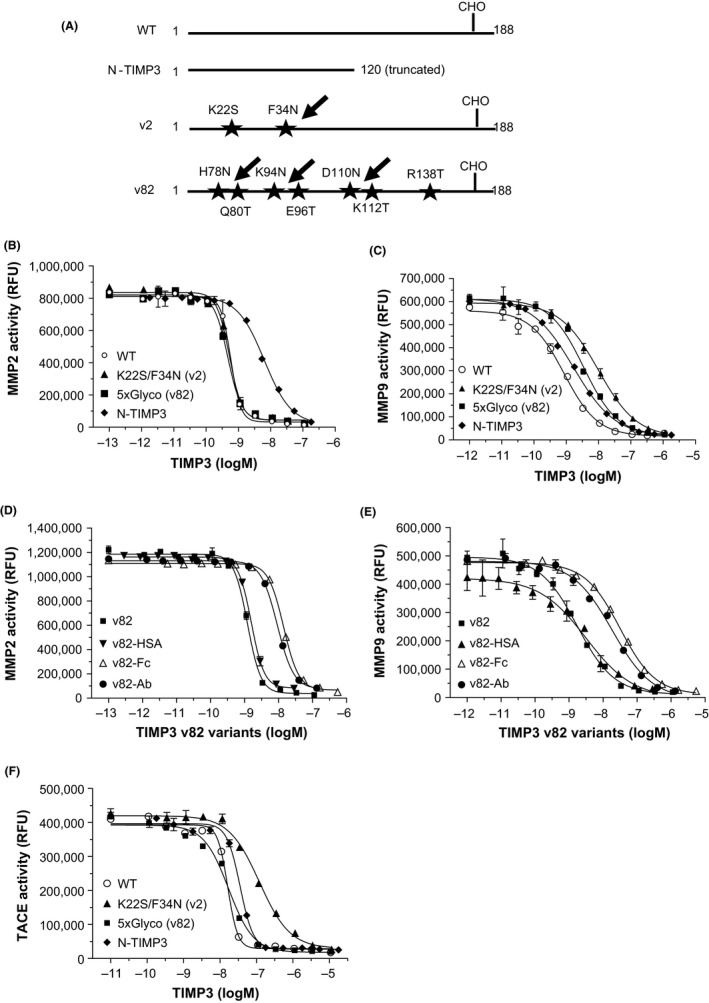
Inhibitory activity of human MMP2, MMP9, and TACE. (A) Schematic diagrams of TIMP3 variant constructs. WT has an endogenous glycosylation site at Asn184 and labeled as CHO. Arrows indicate additional glycosylation sites. N‐TIMP3 was truncated in the C‐terminal domain and does not contain any glycosylation sites. (B and C) Inhibition dose response of human recombinant MMP2 an MMP9 for WT, K22S/F34N (v2), 5x glycosylated (v82) and N‐TIMP3. (D and E) Inhibition dose response of v82 fusion constructs (Fc, HSA, and Ab). (F) Inhibition dose response of human recombinant TACE for WT, K22S/F34N (TIMP3v2), 5x glycosylated (TIMP3v82), and N‐TIMP3. All reactions were for 90 minutes at 37°C incubation. Representative graphs are from n = 3 to 37 experiments. Data are means ± SD

Wild‐type TIMP3 has a low expression yield and short plasma half‐life due to high clearance rates compared to a recombinant antibody protein. In order to overcome these hurdles, TIMP3 was engineered with fusion, glycosylation, PEGylation, and truncation approaches. In general, C‐terminal fusion of HSA, Fc, or antibody heavy chain or light chain improved expression in mammalian cells compared to TIMP3 alone although molecular bulkiness led to a decreased MMP inhibitory potency approximately 2‐5 folds (Figure 1). LRP‐1 is a well‐known scavenger receptor for TIMP3 clearance, and Lysine at amino acid 22 was predicted to interact with LRP‐1. Thus, this residue was mutated (K22S) with additional glycosylation site (F34N), which was confirmed by mass spectrometry analysis (>95% occupancy, data not shown). This construct (K22S/F34N) has significantly improved expression levels (~200 mg/L) compared to the wild type TIMP3 (~10 mg/L), with the K22S/F34N variant (v2) retaining MMP2 potency and approximately 7‐fold reduced potency in MMP9 subtype without dramatic increase in rat serum half‐life (1.4‐fold) (Table 1). The loss of MMP9 potency was more dramatic in rat MMP9 subtype, maybe due to a lower amino acid homology (75%) between human and rat MMP9 compared to MMP2 homology (95%). To reduce potential clearance, we introduced additional glycosylation sites (H55N/Q57T/K71N/E73T/D87N/K89T/R115T, v82) based on structural prediction of LRP‐1 binding sites which extended serum half‐life (4.7‐fold) compared to the wild‐type TIMP3 with retained MMP2/9 inhibitory potency. Site‐specific glycosylation was confirmed by mass spec analysis (data not shown). For v2 and v82 constructs, further TIMP3 C‐terminal fusion with HSA, Fc or antibody heavy chain, and light chain facilitated extending serum half‐life dramatically, especially with Fc fusion (16‐23 hours) although MMP9 potency was lower than the parent molecules. PEGylation of TIMP3v2 with 20 kDa polyethylene glycol (PEG) further increased serum half‐life approximately 25‐fold against the parent molecule. Potency against TACE, in general, decreased with glycosylation or C‐terminal fusion schemes (Table 1). TIMP3 consists of two domains, N‐ (aa 1‐120) and C‐domain (aa 121‐188), with N‐domain known as an efficient pharmacophore from activity measurement and structural analysis.^26^, ^27^ N‐TIMP3, truncated C‐domain, is confirmed active, but exhibited lower MMP2 potency (10‐fold) compared to the full length TIMP3 although MMP9 and TACE potency remained unchanged.

**Table 1:** Summary of MMP inhibition potency (IC_50_) and serum half‐life of engineered TIMP3 molecules

TIMP3	Human IC_50_ (nM ± SD)		Rat IC_50_ (nM ± SD)		Human IC_50_	Rat serum
Variants	MMP2	MMP9	MMP2	MMP9	TACE (nM)	*T*_1/2_ (min)
WT	0.6 ± 0.4	0.9 ± 0.8	1.4 ± 1.3	0.9 ± 0.6	59 ± 52	48
N‐TIMP3 (N)	5.8 ± 3.4	1.5 ± 1.2	3.4 ± 2.1	2.4 ± 2.1	59 ± 48	48
TIMP3v2 (v2)	0.9 ± 0.5	6.9 ± 6.0	0.5 ± 0.4	15.5 ± 10.9	341 ± 189	66
TIMP3v82 (v82)	1.0 ± 0.5	2.3 ± 1.8	0.2 ± 0.1	14.3 ± 9.0	29 ± 11	226
WT‐HSA	0.8 ± 0.9	0.6 ± 0.6	0.2 ± 0.1	50.8 ± 20	12.1 ± 7.6	—
N‐HSA	6.0 ± 4.1	1.1 ± 0.9	3.6 ± 1.0	10.8 ± 4.3	195 ± 164	336
v2‐HSA	5.1 ± 2.1	16 ± 5.8	0.7 ± 0.3	65 ± 28	6800 ± 5200	162
v82‐HSA	1.6 ± 0.7	7.4 ± 3.5	0.3 ± 0.1	152 ± 99	145 ± 104	720
N‐Fc	9.7 ± 1.6	1.6 ± 1.0	—	—	—	—
v2‐Fc	0.9 ± 0.4	10 ± 5.1	0.9 ± 0.3	36 ± 16	618 ± 540	1390
v82‐Fc	2.7 ± 0.4	10.5 ± 8.6	1.0 ± 1.1	77 ± 39	156 ± 85	960
v2‐HC Ab	2.2 ± 0.6	8.6 ± 0.3	0.5 ± 0.6	47 ± 24	—	148
v2‐LC Ab	0.7 ± 0.3	7.4 ± 5.7	0.6 ± 0.8	83 ± 50	—	454
v82‐Ab	8.2 ± 1.0	22.5 ± 9.8	0.9 ± 0.2	181 ± 89	159 ± 20	930
N‐PEG	9.8 ± 4.2	0.7 ± 0.5	1.7 ± 0.1	1.3 ± 1.1	197 ± 118	14
v2‐PEG	0.4 ± 0.2	9.0 ± 4.4	0.1 ± 0.1	60 ± 52	123 ± 7	1716
GM6001	1430 ± 1347	1404 ± 904	803 ± 106	439 ± 354	>10 000	—
PD166793	41 ± 30	1149 ± 945	24 ± 7.7	538 ± 537	>10 000	—
Marimastat	0.8 ± 0.4	1.3 ± 1.1	—	—	1.5 ± 0.4	—

Inhibition dose response of human and rat recombinant MMP2 and MMP9 for WT, K22S/F34N (TIMP3v2), 5x glycosylated (TIMP3v82), N‐TIMP3, fusion constructs (Fc, HSA, and Ab) and PEG molecules. Data are average of n = 3‐37 experiments (mean ± SD). Serum half‐life was determined from Sprague Daley rats after intravenous bolus injection (n = 3). Data are mean ± SD

### 3.2 Zymography of TIMP3 variants

**Figure 2:**
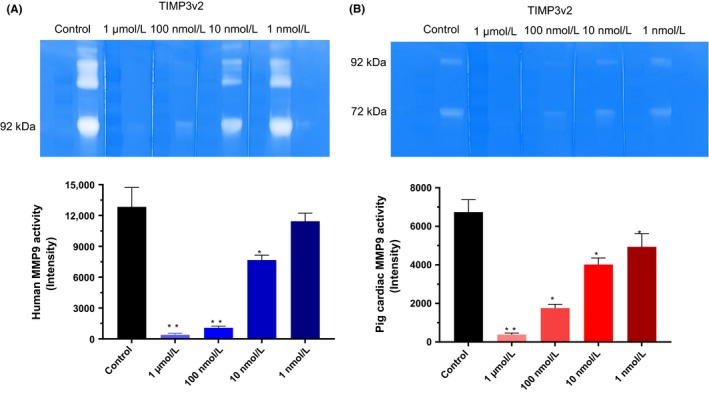
TIMP3 activity in Zymography. (A) Recombinant human MMP9 was run on Gelatin Zymogram. TIMP3v2 (K22S/F34N) was incubated with Developing buffer overnight after cutting gel lanes and transferring into an individual container. After staining and destaining, the gel was quantified using ImageJ software (lower graph). The gel is a representative of n = 3 independent experiments (mean ± SEM). (B) TIMP3v2 (K22S/F34N) inhibition of cardiac tissue MMP activity on the Zymogram from pig cardiac lysates (50 μg/lane) after 4.5 hours post‐MI. Cardiac tissue was homogenized in RIPA buffer with PMSF. After staining and destaining, the gel was quantified using ImageJ software (lower graph). The gel is a representative of n = 3 experiments (mean ± SEM). Graphs are averages of each ImageJ scan. *P <* *0.05 (vs control), **P <* *0.01 (vs control)

The MMP activity of gelatin digestion was detected by unstained intensity of bands on the zymogram gel. TIMP3v2 (K22S/F34N) concentration dependently inhibited the digestive activity of MMP9 on the Zymogram (Figure 2A). Multiple bands are attributed to the multimerization or aggregation of MMP9 because the purified recombinant human MMP9 which was loaded on the zymogram as purified recombinant human MMP9. Due to smears with rat cardiac lysates, we used more reliable pig cardiac lysates. The MMP inhibitory activity was confirmed with the cardiac lysates from the LV infarct area of pig heart 4.5 hours post‐MI (Figure 2B). Upper bands represent an endogenous MMP9 (92 kDa) and the lower bands represent MMP2 (72 kDa) from the cardiac tissue. At a saturating concentration (1 μmol/L), engineered TIMP3 variants inhibited similar MMP activity in the zymogram except some fusion constructs that showed low activity in the gel thought to be due to limited diffusion of bulky molecules (data not shown). The rank order of MMP inhibition in the zymogram was WT‐TIMP3, N‐TIMP3 and TIMP3v2 >  TIMP3v82, and fusion constructs.

### 3.3 Cardiac and plasma half‐life of TIMP3 variants

**Figure 3:**
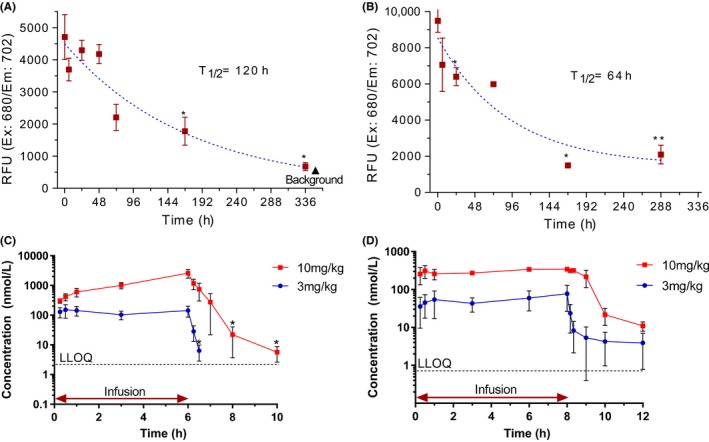
Cardiac and plasma half‐life of TIMP3. (A) Alexa Fluo 680‐labeled TIMP3 (AF680‐K22S/F34N TIMP3, v2, 2 mg/rat) and (B) AF680‐N‐TIMP3 (1 mg/rat) were delivered directly into rat myocardium after 3 hours MI, and hearts were harvested at desired time points postinjection (n = 3 per time point). *P <* *0.05 (vs control), **P <* *0.01 (vs control). (C and D) Rat plasma half‐life of K22S/F34N TIMP3 (C) and N‐TIMP3 (D) after 6 hours continuous jugular vein infusion in 3 mg/kg/h and 10 mg/kg/h before collecting blood for 4 hours postdose (n = 3 per dose). Data are means ± SEM, *P <* *0.05 (vs control), LLOQ, lower limit of quantitation

Exogenously delivered recombinant TIMP3 has a short plasma half‐life. It was not clear if the high clearance is target‐mediated or fast systemic clearance. The clearance pattern of TIMP3 molecules seemed to be much slower in the cardiac tissue after local injection than intravenously delivered TIMP3 (Figure 3). Alexa Fluor labeled TIMP3 (AF680‐TIMP3) molecules were delivered directly into rat myocardium after 3 hours post‐MI, and hearts were harvested at desired time points postinjection (Figure 3A,B). The slower clearance of locally injected TIMP3 molecules could be due to the high affinity of TIMP3 molecules to ECM proteins as well as interaction with elevated MMP enzyme targets in the LV infarct area. The estimated cardiac tissue half‐life was 120 and 64 hours for TIMP3v2 (K22S/F34N) and N‐TIMP3 molecules, respectively. As shown in Table 1, the plasma half‐life of both N‐TIMP3 and TIMP3v2 was <66 minutes in the rat. We hypothesized that with a specific target mediated clearance, a continuous infusion may saturate the clearance target system. Thus, a continuous intravenous infusion of TIMP3 molecules for 6‐8 hours was explored in normal rats (Figure 3C,D). The continuous infusion did not alter the fast clearance kinetics of TIMP3 at least in these rats although nonlinear PK pattern was observed between the low and high dose of TIMP3v2. A nonlinear PK was observed for TIMP3v2 between the low‐ and high‐dose conditions. Both N‐TIMP3 and TIMP3v2 displayed high clearance after systemic administration (Table 2).

**Table 2:** Rat PK profiles of TIMP3 molecules. N‐TIMP3 and TIMP3v2 (K22S/F34N) were continuously infused for 6‐8 h (3 and 10 mg/kg/hr) via jugular vein in Sprague Daley rats (n = 3 per group)

TIMP3	Dose (mg/kg/h)	nmol/kg/h	Concentration^a^ (nmol/L)	Clearance (L/h/kg)	*T*_1/2_ (h)
N‐TIMP3	3	215	82 ± 59	4.27 ± 1.95	2.73 ± 0.77
	10	720	382 ± 20	1.97 ± 0.16	0.66 ± 0.02
TIMP3v2	3	140	132 ± 57	1.28 ± 0.21	0.12 ± 0.01
	10	460	2552 ± 598	0.35 ± 0.07	0.68 ± 0.16

Blood was collected during infusion and 4 h after infusion. Plasma TIMP3 levels were quantified in ELISA. Concentration and *T*_1/2_ data are expressed as the mean ± SEM.

Plasma TIMP3 concentrations at terminal infusion points.

### 3.4 Cardiac functional effects in MI rats

**Figure 4:**
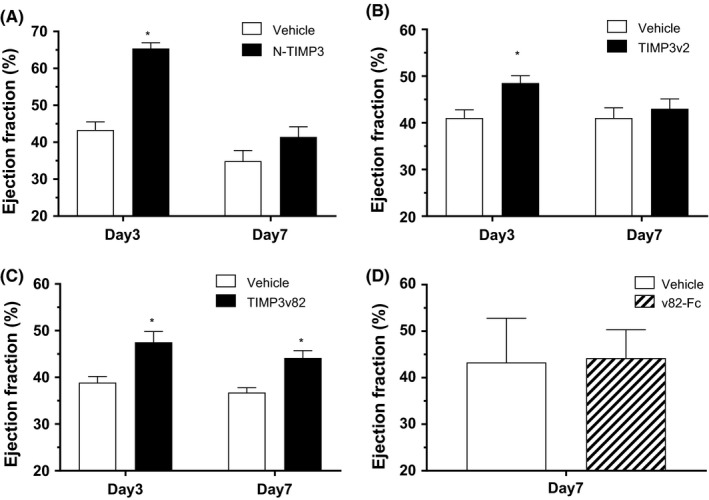
Echocardiography of LAD ligated MI rats. (A‐C) Intramyocardial injection; (A) N‐TIMP3 (1 mg/rat) was injected to directly to the myocardium via 31G needle after 3 hours LAD ligation in SD rats (n = 8). Echocardiography was performed by long axis scanning at Day 3 and Day 7 post‐MI condition. (B) TIMP3v2 (2 mg/rat) was injected directly to the myocardium after 3 hours LAD ligation in SD rats before echocardiography at Day 3 and Day 7 postinjection (n = 8‐10). (C) TIMP3v82 (2 mg/rat) was delivered via tail vein injection after 3 hours LAD ligated MI rats before echocardiography at Day 3 and Day 7 postinjection (n = 8). *P <* *0.05 (vs control), two‐tailed *t* test. (D) No difference was observed with longer lasting TIMP3v82‐Fc infusion. v82‐Fc (5 mg/kg, single IV bolus injection) was infused via tail vein injection post‐LAD ligation (3 hours) in SD rats before echo scanning (n = 8‐9 per group) at Day 7. Data are mean ± SEM

To evaluate the biological activity of TIMP3 as a heart failure target, we characterized N‐TIMP3 (N‐domain, 13.9 kDa), TIMP3v2 (K22S/F34N), 5xGlyco‐TIMP3 (v82), and v82 with Fc fused in the C‐terminus (v82‐Fc). In vitro MMP inhibitory potency was not dramatically different among modified TIMP3 molecules as these molecules potently inhibited MMP2/9 and TACE activities although they showed relatively short IV PK profiles (*t*_1/2_ < 1 hour) except v82‐Fc (*t*_1/2_ = 15 hours). Since cardiac clearance of AF680‐labeled TIMP3 was slower (*t*_1/2 _= 2.5‐5 days) from direct myocardial delivery in MI rats, local delivery of TIMP3 molecules may inhibit MMP activity and retain higher cardiac function in terms of ejection fraction (EF) measurement (Figure 4A‐C). We routinely observed that the local myocardial delivery of TIMP3 molecules effectively increased EF (~10% over control) in the acute stage (3‐4 days) of cardiac remodeling in the rat MI model. Local delivery of TIMP3 variants maintained cardiac function by reducing adverse remodeling in terms of left ventricular end diastolic volume (LVEDV) reduction (Table 3A). This is in line with the data from the pig study, in which TIMP3 inhibition of cardiac remodeling was demonstrated by reducing LVEDV and LV wall thinning in the pig MI model.^31^ A highly glycosylated Fc fused TIMP3 (v82‐Fc) exhibited longer half‐life (15 hours) in the rat after IV delivery, which is suitable for bi‐weekly injection scheme. Thus, v82‐Fc was explored in the rat MI model via tail vein injection. Unexpectedly, twice a week delivery (5 mg/kg) was not different from the untreated control group (Figure 4D).

**Table 3:** Summary of rat cardiac function by intramyocardial delivery of TIMP3 molecules

Dose	PBS	N‐TIMP3	PBS	TIMP3v2	PBS	TIMP3v82
(mg/heart)	—	1	—	2	—	2
@ Day 3						
EF (%)	43.3 ± 2.2	65.4 ± 1.6	38.2 ± 4.5	48.5 ± 1.6	38.9 ± 1.3	47.5 ± 2.3
LVEDV (μL)	287 ± 18	358 ± 36	296 ± 19	279 ± 13	306 ± 16	281 ± 13
LVESV (μL)	162 ± 9	127 ± 9	182 ± 10	144 ± 11	186 ± 9.6	150 ± 13
@ Day 7						
EF (%)	38.9 ± 1.3	41.4 ± 2.7	41 ± 2.2	43 ± 2.1	36.7 ± 1.1	44.1 ± 1.6
LVEDV (μL)	399 ± 21	375 ± 19	398 ± 25	317 ± 15	393 ± 21	310 ± 8
LVESV (μL)	263 ± 11	218 ± 14	236 ± 21	179 ± 10	249 ± 16	173 ± 7
n	8	8	8	10	8	9

As shown in Figure 4A‐C), N‐TIMP3 (1 mg/rat), TIMP3v2 (2 mg/rat) and TIMP3v82 (2 mg/rat) were injected directly into myocardium after 3 h post‐LAD ligation in Sprague Daley rats (n = 8‐10 per group). Echocardiography was performed at Day 3 and Day 7 postdose. %EF was calculated based on the equation: (LVEDV‐LVESV)/LVEDV × 100 (mean ± SEM). Baseline EF at pre‐MI condition was 70 ± 5%

### 3.5 Retention of ECM binding by engineered TIMP3 variants

TIMP3 has been known to bind to the ECM with high affinity and decreased levels associated with after myocardial infarction. To study the ECM binding capacity of the engineered TIMP3 variants compared to WT‐TIMP3, we performed in vitro assays with decellularized ECM. Decellularized ECM was prepared by culturing human HCF in 96‐well plates for a length of time sufficient to produce an ECM layer, followed by cell removal. Time‐lapse assembly of decellularized matrix exhibited increase in fibronectin deposition with culture time until Day 10 when the cells became over‐confluent (data not shown). The fibronectin content in decellularized ECM increased rapidly for wells with high seeding density of HCF compared to longer lag time for sparsely seeded wells.

**Figure 5:**
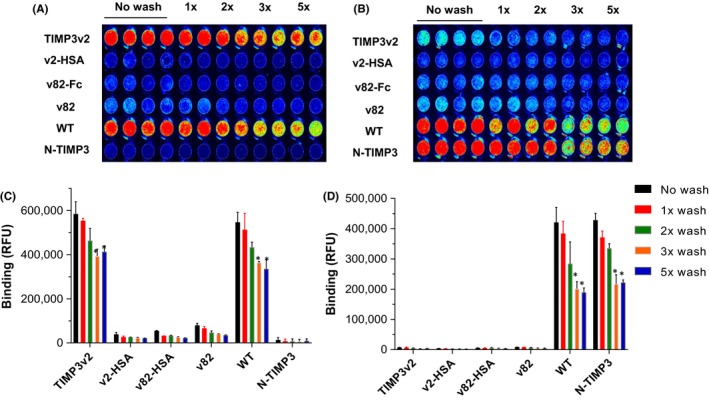
Binding capacity of TIMP3 to acellular ECM. (A,B) TIMP3 molecules were incubated with ECM in 96‐well and washed with PBS and incubated with C‐terminal anti‐TIMP3 antibody (A) and N‐terminal anti‐TIMP3 antibody (B), and the bound protein was detected by IR800‐conjugated secondary antibodies and near IR imaging. After repeated washing with PBS, bound TIMP3 in duplicate wells was determined by near‐IR Odyssey LiCor image. (C,D) No wash includes without wash/fixed and aspirate (duplicates per wash condition). (C) The average of signal intensity for each treatment condition (n = 3). Data are means ± SEM, *P <* *0.05 (vs no wash control), paired *t* test

The ECM binding affinity was assessed with various TIMP3 molecules, TIMP3v2 and N‐TIMP3 showed higher binding than TIMP3‐HSA fusion or multiple sites glycosylated TIMP3 molecules in washout experiments. Since conjugation of IR dyes may alter ECM binding or bioactive site in proteins, we employed immunostaining of unlabeled TIMP3 as a complementary method to measure relative binding affinity of TIMP3 variants after repeated washing with PBS (Figure 5). Out of the TIMP3 variants tested, TIMP3v2 and truncated N‐ TIMP3 displayed significantly higher affinity for decellularized ECM compared to TIMP3‐HSA and glycosylated TIMP3. Immunostaining for TIMP3 with C‐terminal specific antibody (Figure 5A) and N‐terminal specific antibody (Figure 5B) indicates that the ECM binding takes place with WT, TIMP3v2, and N‐TIMP3. Conversely, bulky variants of TIMP3 including fusion and high glycosylation did not exhibit good ECM binding.

**Figure 6:**
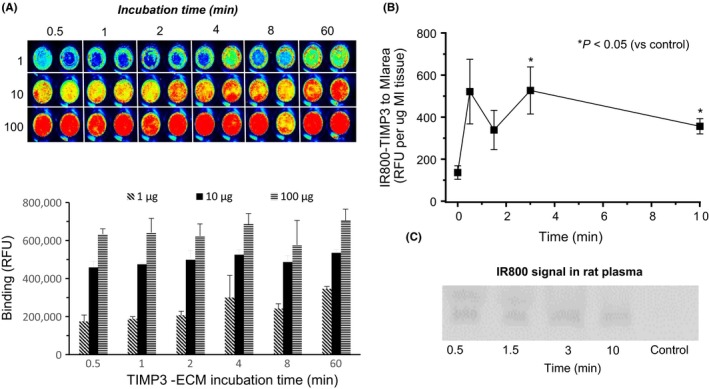
Binding kinetics of TIMP3. (A) TIMP3v2 was incubated with ECM and washed with PBS. ECM bound TIMP3 was stained with anti‐TIMP3 Ab and bound protein was detected by IR800 conjugated secondary antibody and near IR imaging (duplicates wells). (B) Time course of IR800‐labeled TIMP3v2 (1 mg/kg, tail vein) accumulation into the left ventricular infarct area in rat MI hearts (3‐week post‐MI, n = 3 per time point). *P <* *0.05 (vs control), paired *t* test. (C) Plasma levels of IR800‐TIMP3v2 were detected in SDS‐PAGE gel at 0.5, 1.5, 3, and 10 minutes postinjection

IR800‐labeled TIMP3 proteins incubated with ECM showed a concentration dependent increase in retention (Figure 6A). TIMP3 molecules exhibited surprisingly fast ECM binding kinetics as early as 30 seconds in the decellularized ECM condition as well as binding to the infarcted cardiac tissue area in rat hearts post‐MI. IR800‐labeled TIMP3v2 was incubated with ECM and showed a concentration dependent increase in retention after repeated washing with PBS (Figure 6A). A higher concentration induces a stronger signal. At high concentration (100 μg per well), it could bind available ECM binding sites in a saturable manner up to 60 minutes. The binding kinetics was also assessed in the infarct area of MI rat hearts (Figure 6B). An intravenous delivered IR800‐TIMP3v2 was detected in the infarct region as early as 30 seconds, which is technically the earliest time point possible to capture in this in vivo experimental setting. However, the signal increase was plateaued up to 10 minutes after administering through tail vein injection, which may be due to high clearance of the molecule (Figure 6B). The plasma protein level was also detected by gel electrophoresis after blood collection (Figure 6C). This fast localization may be due to higher levels of MMP targets in the infarct area.

### 3.6 Inhibition of ECM degradation by TIMP3

**Figure 7:**
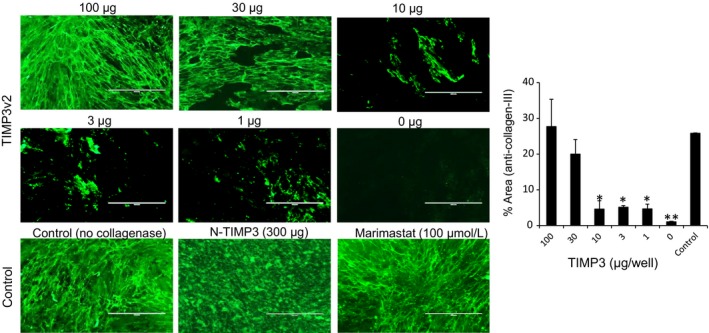
TIMP3 inhibition of collagen fibril degradation by collagenase: Collagenase type‐I (100 μg/well) was incubated with acellular ECM in the presence of varying concentrations of TIMP3v2 (0, 1 μg, 3 μg, 10 μg, 30 μg, 100 μg), N‐TIMP3 (300 μg) or MMP inhibitor Marimastat (100 μmol/L) for 1 hour at 37°C with gentle shake before fixing with 4% PFA. Collagen fibril degradation was assessed by immunostaining using anti‐Collagen type III antibody. Scale bar = 200 μm. Graph is the average of n = 3 experiments with SEM. *P <* *0.05 (vs control), **P <* *0.01 (vs control), paired *t* test

**Figure 8:**
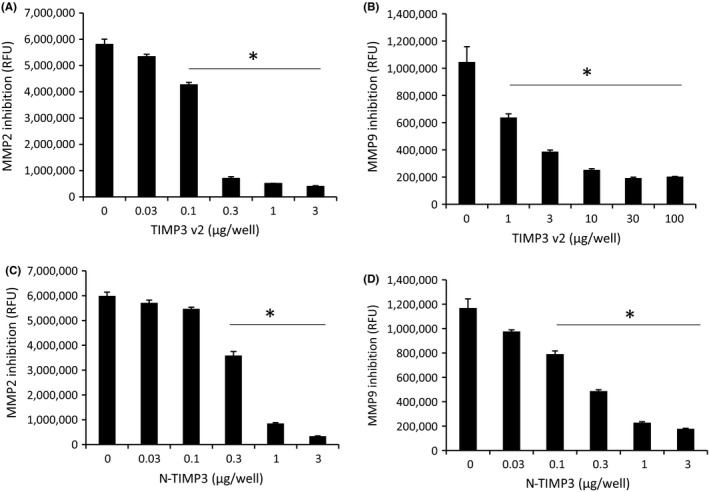
Bioactivity of ECM bound TIMP3. Varying concentrations of TIMP3 were incubated with ECM and washed repeatedly with PBS to remove unbound. Inhibition of MMP2 and MMP9 enzyme activity was assessed by FRET‐based assay for the ECM bound TIMP3v2 (A and B) and N‐TIMP3 (C and D). Graphs are the average of n = 3 experiments (mean ± SEM), *P <* *0.05 (vs control), paired *t* test

In order to detect ECM degradation, the decellularized ECM produced from cardiac fibroblasts was treated with type 1 collagenase. As expected, the ECM was degraded upon collagenase treatment when collagen fibril degradation was assessed by immunostaining with type 3 collagen antibody. TIMP3 displayed a concentration‐dependent inhibition of collagen fibril degradation, similar to that exhibited by small molecule inhibitor marimastat (Figure 7). The biological activity of TIMP3 bound to ECM is dependent on the orientation and accessibility of the bioactive site for interaction with catalytic domain in MMPs. Hence, we assessed the bioactivity of ECM bound TIMP3 by a FRET based assay using fluorescence quenching substrate for MMP9 and MMP2. It demonstrated a concentration‐dependent inhibition of degrading activity by ECM bound TIMP3 (Figure 8). It appears that the ECM bound TIMP3 molecules retained the ability to inhibit MMP2 and MMP9 activity.

**Figure 9:**
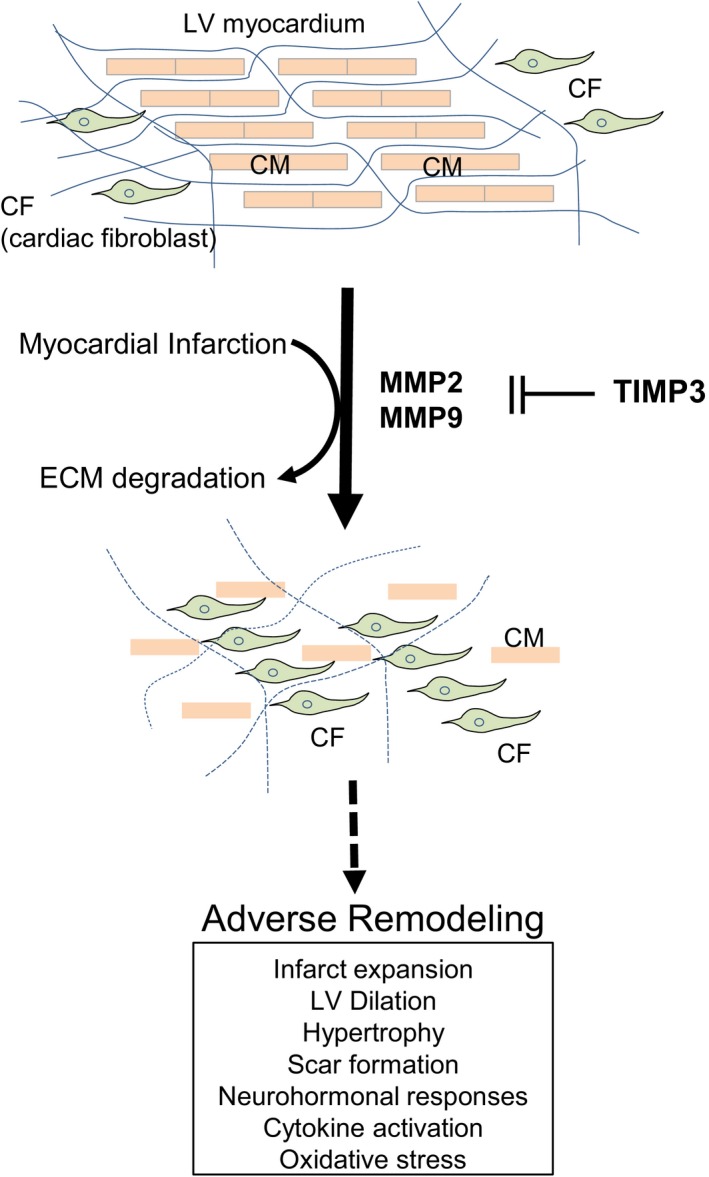
Working hypothesis of TIMP3 in the acute heart failure. MMP and TIMP3 levels are balanced in normal condition. In normal condition, cardiomyocytes (CM) and cardiac fibroblasts (CF) are spatially well organized in the myocardium. Upon ischemic insults, loss of cardiomyocytes is followed by ECM degradation by elevated MMPs. Eventually, myofibroblasts will trigger fibrosis in the infarcted area that leads to adverse remodel in the heart

## 4 DISCUSSION

TIMP3 may have a therapeutic potential to prevent adverse remodeling after myocardial infarction as demonstrated by the beneficial effects of direct TIMP3 injection into the infarcted myocardium in a pig MI model.^31^ This approach could be beneficial for the patients who may undergo the coronary artery bypass graft (CABG) surgery. However, there is a limitation of direct myocardial delivery application because it requires an open chest surgical procedure. Recently, we have explored an intracoronary TIMP3 delivery route via intracoronary catheter in a pig balloon occlusion model, and demonstrated that the intracoronary delivered TIMP3 can prevent adverse myocardial remodeling in ischemia‐reperfusion injuries.^32^ To identify a suitable TIMP3 form to improve biological property and function, we have generated multiple glycosylated forms of TIMP3 and fusion constructs. PEGylation with varying sizes of polyethylene glycols (2‐20 kDa) was employed to many pharmaceutical molecules to improve the pharmacokinetic property as it prevents a rapid clearance due to its bulkiness with PEG conjugation. Indeed, PEGylated TIMP3v2 exhibited longer plasma half‐life (>28 hours) compared to other modifications (Table 1). However, some safety concerns were raised for cellular vacuolization even though it has significant pharmacokinetic effect by slowing down clearance.^22^, ^23^ Modification by glycosylation has been well established in large molecule ligands. For example, the PK profile of darbepoetin alfa (DA), glycosylated EPO, was dramatically improved by two additional glycosylation (5‐N‐Glyco and 1‐O‐Glyco) of recombinant EPO molecule.^26^ Even though TIMP3 has an endogenous glycosylation site in the C‐terminus, additional glycosylation by site‐specific engineering may further improve PK property by increasing stability due to reduced protease sensitivity with bulky sugar moiety, which may also reduce potential immunogenicity.^24^, ^25^ It appeared that additional glycosylation preserved MMP inhibitory activity as well as beneficial cardiac effects in MI rats upon direct myocardial injection, but its plasma half‐life extension was minimal. Interestingly, myocardial half‐life after direct injection into the myocardial tissue exhibited at least 30‐fold longer retention than in the plasma. A direct myocardial injection of v2 and v82 variants showed no noticeable differences in terms of cardiac function in the rat MI model evaluation (Table 3). It was of interest to find if a systemic delivery with a single bolus injection is a more favorable delivery route than the myocardial injection. v82‐Fc allowed us to administer intravenous delivery with extended half‐life of 15 hours. However, it did not exhibit any significant improvement in the same rat MI model. We reasoned that molecule's ECM binding property may play a role. A single additional glycosylation (F34N) does not seem to interfere with ECM binding because TIMP3v2 interacted well with the ECM in the ECM binding experiment. On the contrary, v82 and v82‐Fc molecules did not show binding to ECM with high affinity. It seemed the bulkiness or masking of critical amino acid residues by glycosylation or fusion proteins interferes with ECM binding. The ECM binding of TIMP3 is rapid and also accumulates in the infarcted area, which may be triggered by the leaky vessels in the infarcted LV area that allowed the diffusion of the molecule. The ECM binding affinity may play a role in tissue retention. Intravenously delivered IR800‐TIMP3v2 was detected in the infarct region as early as 30 seconds, which is technically the earliest time point to collect the cardiac tissue. This fast binding kinetics might be beneficial for intracoronary delivery of TIMP3 molecule during balloon angioplasty procedures for the treatment of acute myocardial infarction (AMI). Thus, the data are in line with the notion that the enzymatic activity of MMPs degrades the ECM structure and endogenous inhibitor TIMP3 can counteract the degradation by blocking MMP activity since the ECM bound TIMP3 can efficiently inhibit MMP activity.

There are limitations to the study. First, the variability of in vitro fluorescence‐based MMP assays was high although the assay is more sensitive than the conventional zymography. It may be caused by aggregation of recombinant proteins, which can be improved further to reduce the variability. Second, there were relatively smaller animal numbers in the in vivo studies due to large consumption of proteins, especially infusion studies. A longer lasting TIMP3 variant will be required for future studies. Third, although the present study provided potential mechanistic insight regarding the ECM binding property and inhibition of ECM degradation, it remains to be unclear that this in vitro data can be directly translated into in vivo cardiac effects. More mechanism related studies are warranted. In conclusion, these data may suggest that an exogenous delivery of TIMP3 into the infarcted cardiac area can reduce worsening of cardiac function potentially by preventing ECM degradation in the rat heart.

## DISCLOSURE

None declared.
