# Supplementary material for: Utility of Glycosylated TIMP3 molecules: Inhibition of MMPs and TACE to improve cardiac function in rat myocardial infarct model
Source: Pharmacol Res Perspect. 2018 Nov 14;6(6):e00442. doi: 10.1002/prp2.442 (PMC6234480; doi:10.1002/prp2.442)
Supplement: Supplementary file 1 [file PRP2-6-e00442-s001.pptx]

## Slide 1
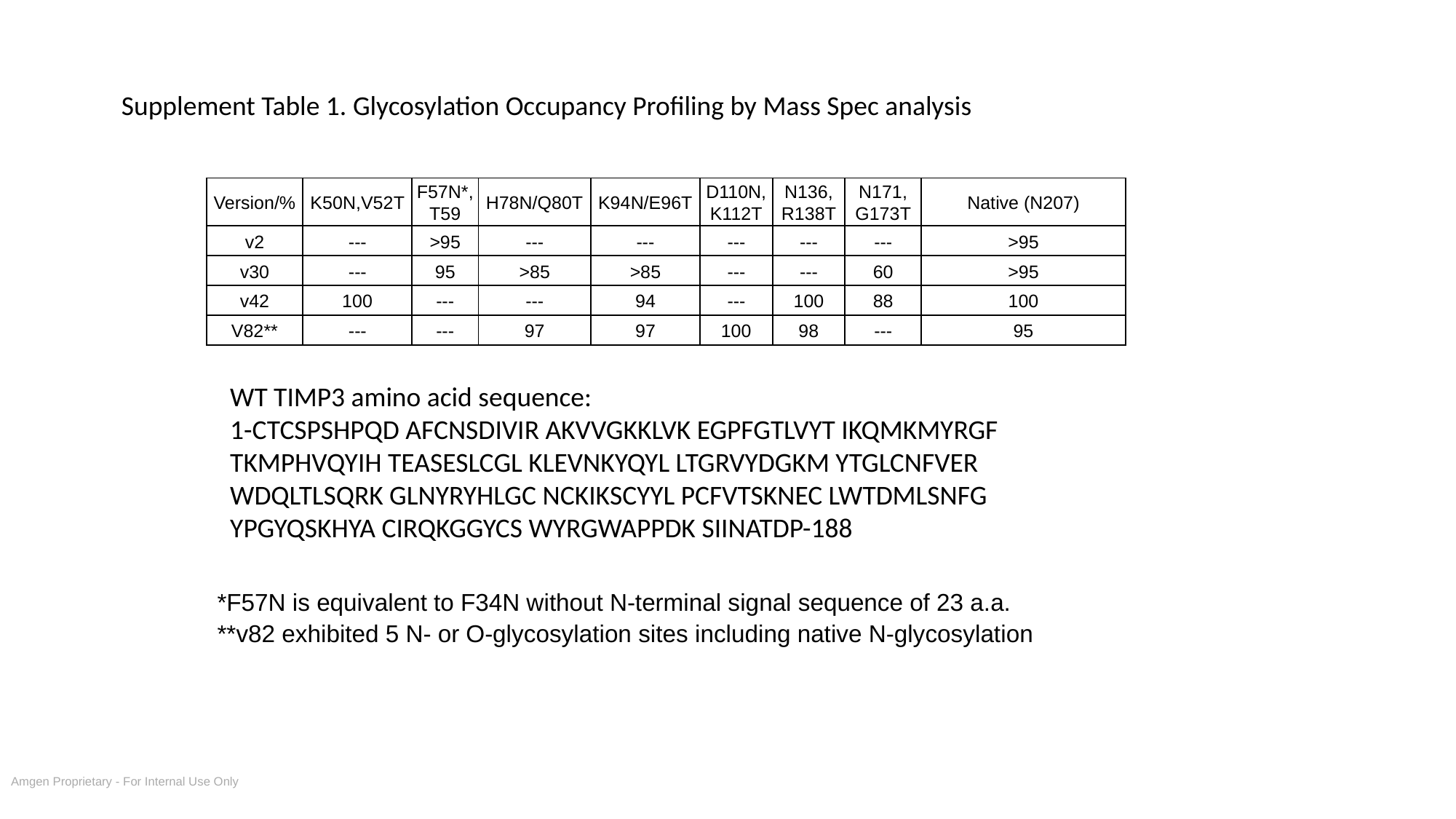

Supplement Table 1. Glycosylation Occupancy Profiling by Mass Spec analysis
| Version/% | K50N,V52T | F57N\*, T59 | H78N/Q80T | K94N/E96T | D110N, K112T | N136, R138T | N171, G173T | Native (N207) |
| --- | --- | --- | --- | --- | --- | --- | --- | --- |
| v2 | --- | >95 | --- | --- | --- | --- | --- | >95 |
| v30 | --- | 95 | >85 | >85 | --- | --- | 60 | >95 |
| v42 | 100 | --- | --- | 94 | --- | 100 | 88 | 100 |
| V82\*\* | --- | --- | 97 | 97 | 100 | 98 | --- | 95 |
WT TIMP3 amino acid sequence:
1-CTCSPSHPQD AFCNSDIVIR AKVVGKKLVK EGPFGTLVYT IKQMKMYRGF
TKMPHVQYIH TEASESLCGL KLEVNKYQYL LTGRVYDGKM YTGLCNFVER
WDQLTLSQRK GLNYRYHLGC NCKIKSCYYL PCFVTSKNEC LWTDMLSNFG
YPGYQSKHYA CIRQKGGYCS WYRGWAPPDK SIINATDP-188
*F57N is equivalent to F34N without N-terminal signal sequence of 23 a.a.
**v82 exhibited 5 N- or O-glycosylation sites including native N-glycosylation
Amgen Proprietary - For Internal Use Only

## Slide 2
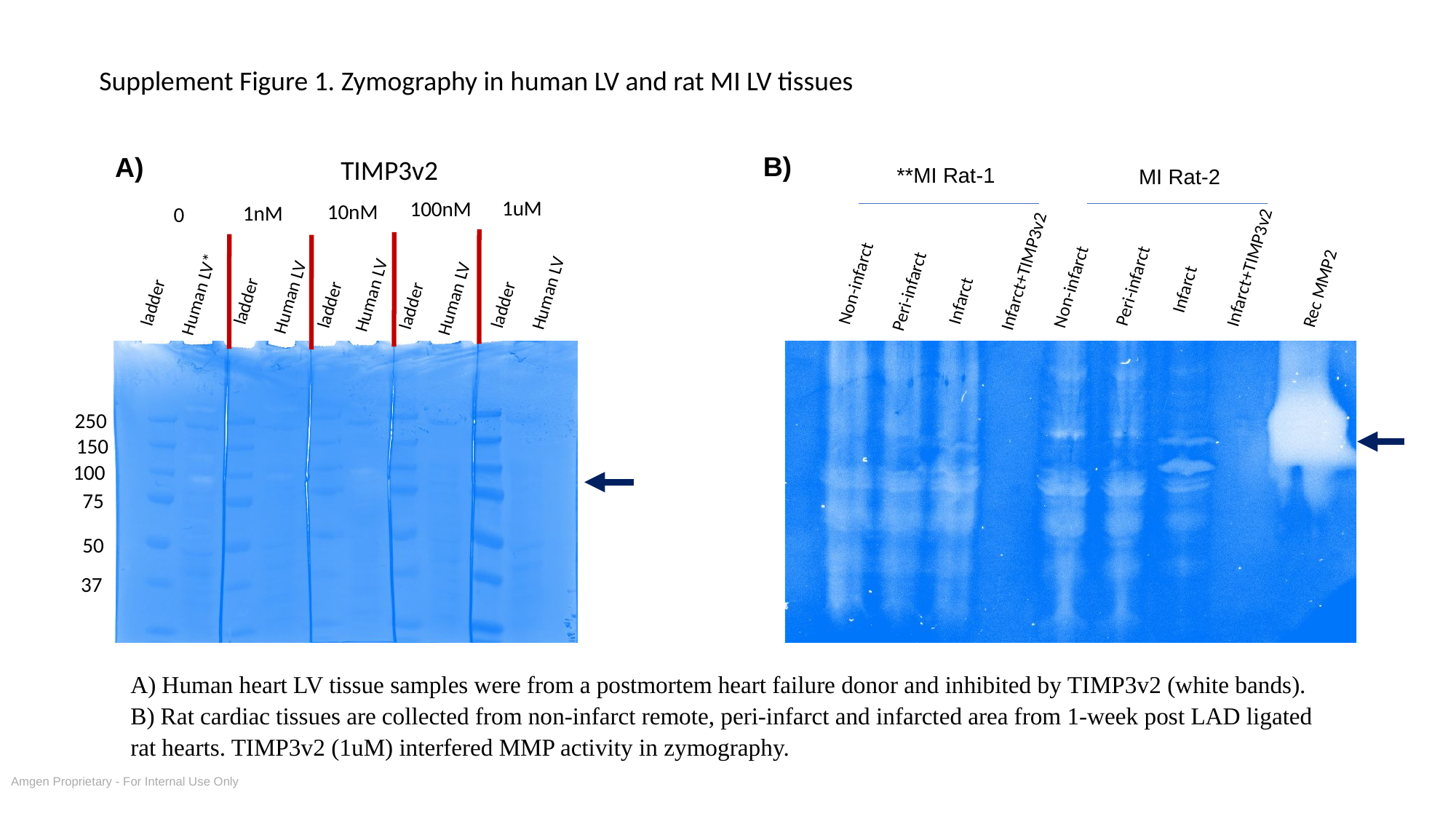

Supplement Figure 1. Zymography in human LV and rat MI LV tissues
**MI Rat-1
MI Rat-2
B)
A)
TIMP3v2
1uM
100nM
10nM
1nM
0
Human LV*
Human LV
Human LV
Human LV
Human LV
ladder
ladder
ladder
ladder
ladder
250
150
100
75
50
37
Infarct+TIMP3v2
Infarct+TIMP3v2
Non-infarct
Non-infarct
Peri-infarct
Rec MMP2
Peri-infarct
Infarct
Infarct
A) Human heart LV tissue samples were from a postmortem heart failure donor and inhibited by TIMP3v2 (white bands).
B) Rat cardiac tissues are collected from non-infarct remote, peri-infarct and infarcted area from 1-week post LAD ligated rat hearts. TIMP3v2 (1uM) interfered MMP activity in zymography.
Amgen Proprietary - For Internal Use Only
